# Performance of urinary C–C motif chemokine ligand 14 for the prediction of persistent acute kidney injury: a systematic review and meta-analysis

**DOI:** 10.1186/s13054-023-04610-7

**Published:** 2023-08-18

**Authors:** Yih‑Ting Chen, Heng-Chih Pan, Cheng-Kai Hsu, Chiao-Yin Sun, Chun-Yu Chen, Yi-Hung Chen, Heng-Jung Hsu, I-Wen Wu, Vin-Cent Wu, Eric Hoste

**Affiliations:** 1 Chang Gung University College of Medicine, Taoyuan, Taiwan; 2 Division of Nephrology, Department of Internal Medicine, Keelung Chang Gung Memorial Hospital, 222 Mai-Jin Road, Keelung, 204 Taiwan; 3 Graduate Institute of Clinical Medicine, College of Medicine, National Taiwan University, Taipei, Taiwan; 4 Community Medicine Research Center, Keelung Chang Gung Memorial Hospital, Keelung, Taiwan; 5 Department of Pharmacy, Keelung Chang Gung Memorial Hospital, Keelung, Taiwan; 6 Division of Nephrology, Department of Internal Medicine, Shuang Ho Hospital, New Taipei City, Taiwan; 7 Taipei Medical University, Taipei, Taiwan; 8 Division of Nephrology, Department of Internal Medicine, National Taiwan University Hospital, Taipei, Taiwan; 9 Intensive Care Unit, Department of Internal Medicine and Pediatrics, Ghent University Hospital, Ghent University, Ghent, Belgium; 10 Research Foundation-Flanders (FWO), Brussels, Belgium

## Abstract

**Background:**

Urinary C–C motif chemokine ligand 14 (CCL14) has been described as an effective marker for delayed recovery of acute kidney injury (AKI), yet its efficacy has been found to vary between different trials. The goal of this research was to assess the predictive performance of urinary CCL14 as a marker for persistent AKI.

**Methods:**

In accordance with the Preferred Reporting Items for Systematic Reviews and Meta-Analyses (PRISMA) guidelines, we searched the PubMed, Embase, and Cochrane databases up to April 2023 for studies of adults (> 18 years) that reported the diagnostic performance of urinary CCL14. The sensitivity, specificity, number of events, true positive, and false positive results were extracted and evaluated. Hierarchical summary receiver operating characteristic curves (HSROCs) were used to summarize the pooled test performance, and the Grading of Recommendations, Assessment, Development and Evaluations criteria were used to appraise the quality of evidence.

**Results:**

We included six studies with 952 patients in this meta-analysis. The occurrence of persistent AKI among these patients was 39.6% (377/952). The pooled sensitivity and specificity results of urinary CCL14 in predicting persistent AKI were 0.81 (95% CI 0.72–0.87) and 0.71 (95% CI 0.53–0.84), respectively. The pooled positive likelihood ratio (LR) was 2.75 (95% CI 1.63–4.66), and the negative LR was 0.27 (95% CI 0.18–0.41). The HSROC with pooled diagnostic accuracy was 0.84.

**Conclusion:**

Our results suggest that urinary CCL14 can be used as an effective marker for predicting persistent AKI.

**Supplementary Information:**

The online version contains supplementary material available at 10.1186/s13054-023-04610-7.

## Background

Acute kidney injury (AKI) is a condition that occurs in 40% to 70% of critically ill patients, and that is associated with worse outcomes such as increased length of stay and costs, morbidity and mortality [1, 2]. Persistent AKI, defined as AKI with a duration of 48 h or longer, is associated with worse outcomes compared to AKI with rapid reversal [3]. The persistence of AKI is a matter of great importance, as it significantly increases a patient’s risk of developing a variety of associated complications such as cardiovascular disease, hypertension, chronic kidney disease (CKD), end-stage renal disease (ESRD), and mortality [4–7]. Early identification of persistent AKI may facilitate timely interventions and so improve outcomes [8–11].

Urinary C–C motif chemokine ligand 14 (CCL14), also known as human CC chemokines-1 (HCC-1), has been identified as a potential biomarker for AKI and may provide new insights into the pathophysiology of AKI [12–14]. CCL14 is a small cytokine belonging to the chemokine family [15, 16]. It is primarily produced by macrophages and monocytes and is thought to play an important role in recruiting immune cells to sites of injury or infection and in regulating inflammation in various organ systems, including the kidney [13]. The RUBY study and subsequent studies demonstrated that CCL14 levels were significantly elevated in the urine of patients with AKI and elevated urinary CCL14 levels were associated with persistent AKI [17–20].

Since these studies included a limited number of patients, a meta-analysis was conducted to evaluate the diagnostic value of urinary CCL14 in predicting persistent AKI.

## Methods

### Data sources and search strategy

**Fig. 1:**
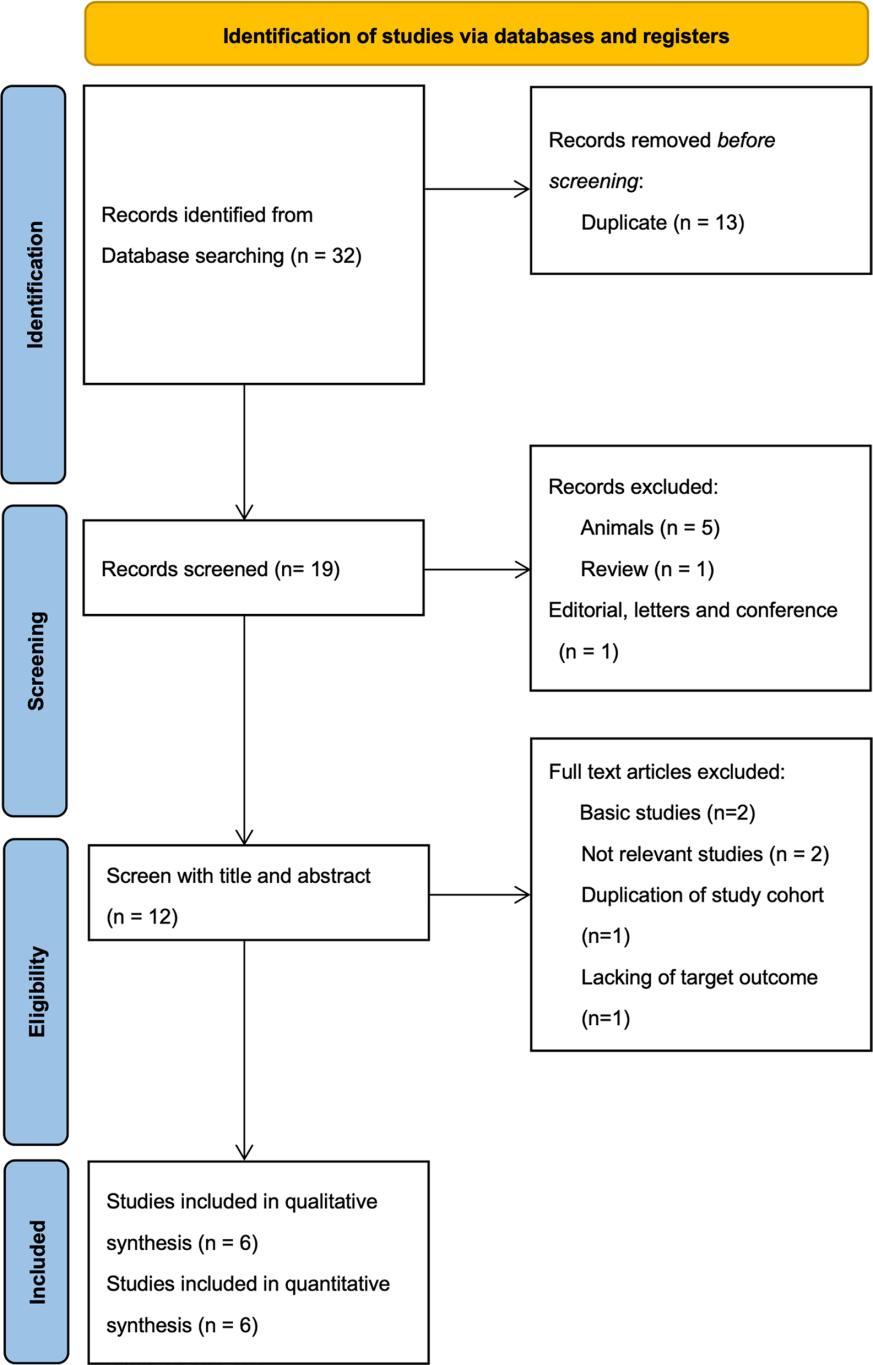
Flowchart of study selection for meta-analysis

The primary outcome was persistent AKI. We performed electronic searches on PubMed, Medline and Embase from inception to April 12, 2023. The search strategies are listed in Fig. 1. We collected information regarding the clinical setting, timing of urinary CCL14 measurement, definition of persistent AKI, patient number, and the number of participating centers (Table 1). We reviewed references by evaluating their titles and abstracts, and selected studies that were relevant for further analysis. We manually checked the reference lists of relevant studies, systematic reviews, and meta-analyses to find any additional publications that could be useful for our analysis. We evaluated both the abstracts and full papers for their quality and included them in our synthesis of data. We also contacted the authors of the abstracts we selected for further details. This systematic review and meta-analysis was carried out following the guidelines outlined in the Preferred Reporting Items of Systematic Reviews and Meta-Analyses (PRISMA) statement, and utilizing the Cochrane methodology. We prospectively submitted the systematic review protocol for registration on PROSPERO [No. CRD42023399055].

**Table 1:** Characteristics of included studies

No	Study	Population setting	Timing of CCL14 measurement	Definition of persistent AKI	Definition of AKI	Total Patient	Distribution of AKI severity among all included patients, %	Persistent AKI, %	Multi/single center
1	Massoth et al. [25]	Cardiac surgery patients with AKI stage 2 or 3	At enrollment	AKI stage 3 ≥ 72 h	KDIGO SCr and UO criteria	100	Stage 2: 83 (83.0%) Stage 3: 17 (17.0%)	37 (37.0%)	Single center
2	Koyner et al. [24]	Critically ill patients with AKI stage 2 or 3	At enrollment	AKI stage 3 ≥ 72 h	KDIGO SCr and UO criteria	335	No AKI: 18 (5.4%) Stage 1: 37 (11.0%) Stage 2: 169 (50.4%) Stage 3: 111 (33.1%)	110 (32.8%)	Multi-nation
3	Pan et al. [26]	Critically ill patients with dialysis-requiring AKI	Before weaning of RRT	RRT dependence at 90 days after discharge	KDIGO SCr criteria	140	Stage 3: 140 (100%)	54 (38.6%)	Multi-center
4	Qian et al. [19]	Critically ill patients with AKI	At enrollment	No renal recovery within 7 days	KDIGO SCr and UO criteria	164	Stage 1: 91 (55.5%) Stage 2: 54 (33.0%) Stage 3: 19 (11.6%)	64 (39.0%)	Multi-center
5	Jiang et al. [18]	Critically ill patients with AKI	At enrollment	AKI stage 2 or 3 ≥ 48 h	KDIGO SCr and UO criteria	18	N/A	9 (50.0%)	Single center
6	Meersch et al. [20]	Critically ill patients with oliguric stage 2 AKI	At enrollment	AKI stage 3 at 90 days	KDIGO UO criteria	195	Stage 2: 92 (47.2%) Stage 3: 103 (52.8%)	103 (52.8%)	Single center

AKI, acute kidney injury; KDIGO, Kidney Disease Improving Global Outcomes; SCr, serum creatinine; UO, urine output

### Inclusion and exclusion criteria

We included studies in our analysis that met the following criteria: (1) studies that enrolled participants who were 18 years or older, (2) studies which reported the use of urinary CCL14 for the detection of AKI, (3) studies that evaluated the incidence of persistent AKI, as defined by the study investigators, (4) provision of comprehensive information, including sample size, sensitivity, and specificity at a designated cutoff value, thereby facilitating the pooling of data for accuracy analysis, and (5) in cases where multiple studies were available from the same database and reported on similar or overlapping patient populations, we selected the most recent publication for analysis.

We excluded studies that met any of the following criteria: letters, conference reports, or case reports. By adopting this selection approach, our aim was to maximize the accuracy and representativeness and avoid potential data duplication in our meta-analysis by incorporating the latest available evidence from the chosen database.

### Study selection and data extraction

Two independent reviewers (YTC and HCP) assessed searched articles, including title, abstract, and full text to determine the eligibility. Divergences were resolved by discussion with a third investigator (VCW). All relevant data were independently extracted from the included studies by two reviewers (YTC and HCP). The data that were extracted included information about the study (such as first author, year of publication, study design, study population, genre of biomarkers and timing of measurements, main study outcome and events) and the patients’ baseline data (such as the mean age, gender, underlying diseases, and the severity of illness).

### Quality assessment

The Quality Assessment of Diagnostic Accuracy Studies-2 (QUADAS-2) tool was used to assess the quality of each included study [21]. The following four domains were assessed: patient selection, index test, reference standard, and flow and timing.

The criteria for rating study quality were as follows: high-risk study (2 or more items rated as high risk of bias); low-risk study (5 or more items rated as low risk and no more than one as high risk); moderate-risk study (all remaining situations) [22, 23].

Any disagreements in the quality assessment were resolved by discussion and consensus.

### Data synthesis and statistical analysis

We calculated the sensitivity, specificity, positive predictive value, and negative predictive value of the cutoff value of urinary CCL14 in predicting persistent AKI using the number of true positives (TP), true negatives (TN), false positives (FP), and false negatives (FN) obtained from the study. If the study only provided sample size, sensitivity and specificity, we used these values to back-calculate the TP, TN, FP, and FN. We then used Stata 14.1 for Mac and the "midas" module to generate a hierarchical summary receiver operating characteristic curve (HSROC) to evaluate the overall diagnostic performance of urinary CCL14. We used funnel plots to check for publication bias. A two-sided *P* value < 0.05 was considered statistically significant.

## Results

### Search results and study characteristics

The study selection process is summarized in Fig. 1. A total of 32 articles were identified through database searching. After removing duplicates, 19 articles were evaluated by their titles and abstracts. Out of these, six studies met the criteria for a full-text review [18–20, 24–26]. During the literature search process, we found that the RUBY study, authored by Hoste et al. [17], and the subsequent secondary analysis article by Koyner et al. [24] shared the same study population. To avoid data duplication, we opted to include the more recent study data from Koyner et al., which also provided the target outcome, for our integrated analysis. From the studies conducted by Jiang et al. and Meersch et al., in order to reduce the research heterogeneity, we extracted data from subpopulations within those two studies [18, 20]. These studies included information on 952 patients, who had data on the incidence of persistent AKI with urinary CCL14 values, and were included in the meta-analysis. Tables 1 and 2 show the details of these studies, as well as the population characteristics and AKI diagnosis definition used. Among the six included studies, four studies exclusively enrolled patients from a mixed ICU, and two studies exclusively enrolled surgical patients. All six studies used the KDIGO classification as the definition for AKI. To be specific, four studies utilized both serum and urine criteria of the kidney disease improving global outcomes (KDIGO) classification, while one study solely relied on serum criteria, and the remaining study solely relied on urine criteria. Of the enrolled study, three studies enrolled patients with moderate to severe AKI (stage 2 or 3), one study only enrolled AKI patients treated with renal replacement therapy (RRT), and the remaining two studies enrolled patients with AKI of any stage. All six studies provided quantifiable results for persistent AKI. Among them, two studies defined persistent AKI as AKI stage 3 persisting for 72 h, one study defined it as AKI stage 2 or 3 persisting for 48 h, one study defined it as AKI stage 3 at day 90, one study defined it as RRT dependence at 90 days after discharge, and one study defined it as the absence of renal recovery within 7 days.

**Table 2:** Summary of patients’ characteristics of included studies

No	Study	Mean age, years	Male gender, %	Baseline SCr, mg/dL	HTN, %	Diabetes, %	Heart failure, %	Sepsis, %	Surgery, %	Disease severity
1	Massoth et al. [25]	72.5	73 (73.0%)	1.0	72 (72.0%)	27 (27.0%)	33 (33.0%)	Unknown	100 (100%)	SOFA 10 APACHE 22
2	Koyner et al. [24]	64	209 (62.4%)	1.0	228 (68.1%)	110 (32.8%)	73 (21.8%)	75 (22.4%)	105 (31.3%)	Non-renal APACHE III 54
3	Pan et al. [26]	61.3	101 (72.1%)	1.84	72 (51.4%)	58 (41.4%)	Unknown	68 (48.6%)	88 (62.9%)	SOFA 10.8
4	Qian et al. [19]	58	102 (62.2%)	0.74	57 (34.8%)	30 (18.3%)	Unknown	43 (26.2%)	112 (68.3%)	Non-renal SOFA 4.8 APACHE II 14.6
5	Jiang et al. [18]	65.5	25 (52.1%)	1.56	26 (54.2%)	13 (27.1%)	Unknown	48 (100%)	Unknown	SOFA 9
6	Meersch et al. [20]	69.5	131 (63.0%)	1.04	142 (68.3%)	53 (25.5%)	51 (24.5%)	Unknown	208 (100%)	SOFA 11.4 APACHE 28.4

APACHE, The Acute Physiology and Chronic Health Evaluation; HTN, hypertension; SCr, serum creatinine; SOFA, sequential organ failure assessment

### Quality of the enrolled trials

All six studies were published in the recent three years [18–20, 24–26]. The included number of patients ranged from 18 to 335. The QUADAS-2 tool revealed that the quality of each study varied. There was a low or unclear risk in each study in most domains of bias evaluation (Additional file 1: Figs. S1–S2). The risk of bias was low for patient selection in five studies (83.3%); index test in one study (16.6%); reference standard in six studies (100%); and flow and timing in three studies (50.0%). The applicability concerns were low for patient selection in one study (16.6%); index test in five studies (83.3%); and reference standard in six studies (100%). Therefore, according to the criteria of overall quality, four studies (66.6%) were rated as low risk, two studies (33.3%) as unclear risk, and none study as high risk.

### Primary outcomes

**Fig. 2:**
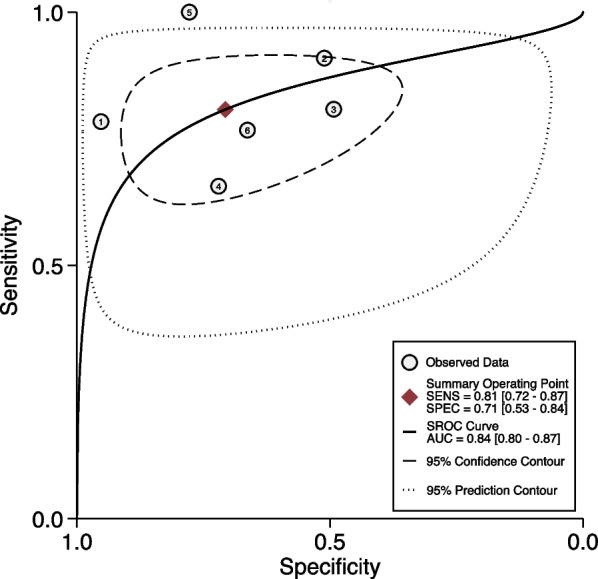
Accuracy of urinary CCL14 for prediction of persistent AKI. HSROC curve with Prediction and Confidence contours. Circles symbolize estimates of individual primary studies, and a square indicates the summary point of sensitivity and specificity. The HSROC curve is plotted as a curvilinear line passing through the summary point. The overall discriminative accuracy of the urinary CCL14 to predict persistent AKI shows an area under the curve (AUC) of 0.84 (0.80–0.87). AKI, acute kidney injury; CCL14, C–C motif chemokine ligand 14; HSROC, hierarchical summary receiver operating characteristic

**Fig. 3:**
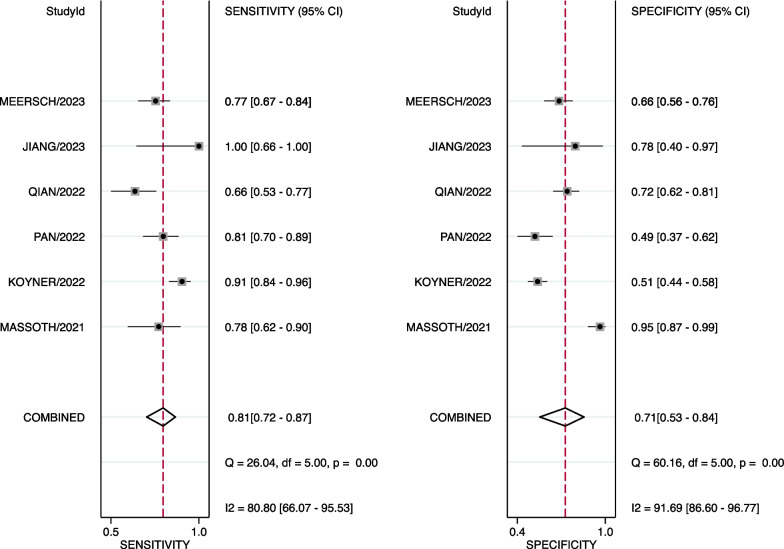
The forest plot for evaluating the pooled sensitivity and specificity of urinary CCL14 in predicting persistent AKI. AKI, acute kidney injury; CCL14, C–C motif chemokine ligand 14; CI, confidence interval

**Fig. 4:**
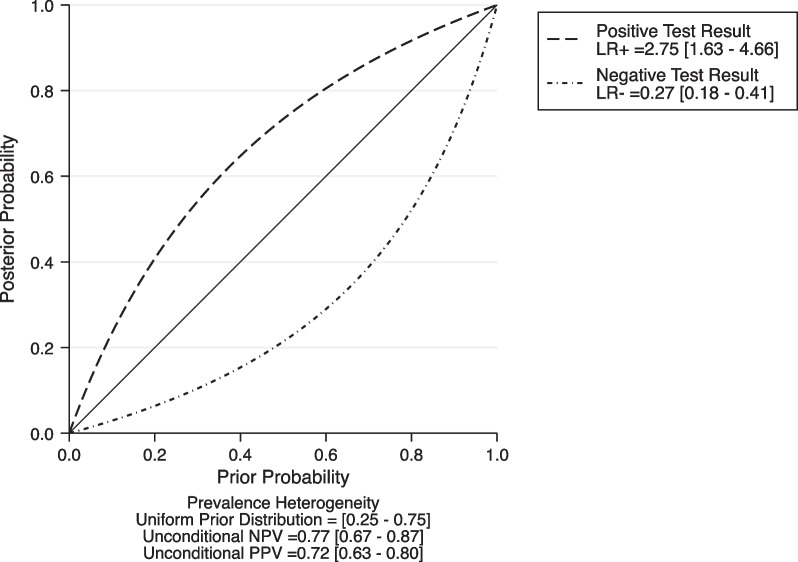
The positive and negative likelihood ratios of urinary CCL14 predictive accuracy for persistent AKI. AKI, acute kidney injury; CCL14, C–C motif chemokine ligand 14; LR, likelihood ratio; NPV, negative predictive value; PPV, positive predictive value

Based on all of included studies with a total of 952 patients, 377 patients (39.6%) had persistent AKI. The diagnostic values, cutoffs, and key results are summarized in Table 3. Among them, the cutoff values of urinary CCL14 range from 0.4 to 2.44 ng/ml, according to different studies. The sensitivity for diagnosing persistent AKI varies from 0.66 to 1.00, while the specificity ranges from 0.49 to 0.95. The numbers of CCL test positives and test negatives are also listed in Table 3, according to the respective studies. The HSROC depicting the overall discriminative accuracy of the urinary CCL14 to predict persistent AKI shows an area under the curve (AUC) of 0.84 (0.80–0.87) (Fig. 2). The forest plot for evaluating the pooled sensitivity of urinary CCL14 for predicting persistent AKI is 0.81 (95% CI 0.72–0.87) and the pooled specificity is 0.71 (95% CI 0.53–0.84) (Fig. 3). Heterogeneity was remarked in the pooled sensitivity (*I*^2^ = 80.80%, *p* < 0.001) and pooled specificity analyses (*I*^2^ = 91.69%, *p* < 0.001). The pooled positive LR is 2.75 (95% CI 1.63–4.66), and the negative LR is 0.27 (95% CI 0.18–0.41) (Fig. 4). The Fagan nomogram discloses that, in the enrolled patient population in the current meta-analysis with a prevalence of persistent AKI of 39.6%, the post-test likelihood increases to 64% when the test is positive. Under the same prevalence, it decreases to 15% when the test is negative (Additional file 1: Fig. S3a). If the prevalence of persistent AKI is 25%, the post-test likelihood increases to 48% when the test is positive. Under the same prevalence, it decreases to 8% when the test is negative (Additional file 1: Fig. S3b). If the prevalence of persistent AKI increases to 75%, the positive post-test likelihood is 89% and the negative post-test likelihood is 45% (Additional file 1: Fig. S3c).

**Table 3:** Diagnostic test performance of CCL14 for persistent AKI

No	Study	Cutoff value of CCL14, ng/mL	Sensitivity	Specificity	Sample size	Event (Persistent AKI), %	CCL test positive number	CCL test negative number	TP	FP	FN	TN	AUC
1	Massoth et al. [25]	2.21	0.78	0.95	100	37 (37.0%)	32	68	29	3	8	60	0.93
2	Koyner et al. [24]	1.3	0.91	0.51	335	110 (32.8%)	210	125	100	110	10	115	0.82
3	Pan et al. [26]	0.4	0.81	0.49	140	54 (38.6%)	93	47	59	34	14	33	0.67
4	Qian et al. [19]	0.626	0.66	0.72	164	64 (39.0%)	70	94	42	28	22	72	0.71
5	Jiang et al. [18]	0.603	1.00	0.78	18	9 (50.0%)	11	7	9	2	0	7	0.85
6	Meersch et al. [20]	2.44	0.77	0.66	195	103 (52.8%)	110	85	79	31	24	61	0.72

AKI, acute kidney injury; APACHE, The Acute Physiology and Chronic Health Evaluation; CCL14, C–C motif chemokine ligand 14; HTN, hypertension; SCr, serum creatinine; SOFA, sequential organ failure assessment; TP, true positive; FP, false positive; FN, false negative; TN, true negative

### Publication bias

Funnel plots were generated to evaluate the possibility of publication bias, which showed generally symmetrical distributions. This result suggests that publication bias is unlikely in this meta-analysis (*p* = 0.37) (Additional file 1: Fig. S4).

### Sensitivity analysis

Among the six studies included, Pan’s study differed significantly in terms of population setting, specifically focused on critically ill patients who are treated with RRT. In contrast, the other five studies involved critically ill patients with AKI, regardless of whether they received RRT or not. However, after excluding Pan’s study, the combined data from the remaining five studies align with our main findings, as depicted in Additional file 1: Figs. S5–S7. Notably, our findings remained consistent and robust in sensitivity analyses, as the results were unaffected by the removal of any individual study (data not shown). This result supports the utility of urinary CCL14 as an effective marker for predicting persistent AKI.

### Assessment of evidence quality and summary of findings

The quality of evidence was assessed using the Grading of Recommendations, Assessment, Development, and Evaluations (GRADE) system. We evaluated the primary outcome and presented them as summary of findings. The certainty of evidence (COE) for the primary outcome was determined to be low due to the observational nature of the included studies. Although there was a low risk of bias, indirectness, imprecision, and publication bias, the COE was downgraded due to inconsistent results among the included studies. For a more comprehensive understanding of our COE assessment, please refer to the Additional file 1.

## Discussion

The current study aimed to summarize the predictive performance of urinary CCL14 for persistent AKI in hospitalized critically ill patients or patients undergoing heart surgery. Nearly one-third of AKI patients experience persistent AKI, highlighting the need for accurate biomarkers to aid in its prediction. To the best of our knowledge, this is the first study to conduct a meta-analysis on the diagnostic accuracy of urinary CCL14 for predicting persistent AKI. A total of six studies, comprising 952 patients, were included in the analysis. The meta-analysis demonstrated that urinary CCL14 has a good overall accuracy in predicting persistent AKI, with an overall discriminatory accuracy of 0.84 (0.80–0.87) (Additional file 1: Fig. S8). The positive and negative likelihood ratios were above 2.5 and below 0.3, further supporting the reliability and accuracy of urinary CCL14 as a biomarker for predicting persistent AKI.

In clinical practice, early detection of persistent and severe AKI may prevent further damage to kidney function as it may trigger the use of the AKI bundle to prevent further deterioration of AKI [27–30]. Patients with severe and persistent AKI have a higher risk of developing CKD and ESRD than those with non-severe and persistent AKI [31–34]. Early diagnosis and intervention may help to reduce mortality rates and the economic burden of healthcare [30, 35]. The 23rd ADQI consensus work group suggests that biomarkers can be combined with traditional methods to stratify risk, distinguish etiologies, assess severity, and predict the duration and recovery of AKI [14]. In particular, urinary CCL14 has emerged as a promising biomarker with the ability to forecast severe and persistent AKI based on previous research [17, 20, 24, 25, 36]. CCL14 is expressed naturally in various tissues [37], including the kidney, in order to recruit immune cells to sites of injury or infection and to regulate inflammatory response. Its involvement in proinflammatory chemotaxis has been documented in several diseases such as multiple myeloma, lupus, hepatocellular carcinoma, inflammatory bowel disease, and rheumatoid arthritis, through the activation of monocytes and macrophages [38–41]. The precise role of urinary CCL14 in AKI is not yet comprehensively understood. It has been proposed that the release of CCL14 from injured renal tubular epithelial cells is stimulated by inflammatory mediators. The binding of CCL14 to receptors on T-cells and monocytes induces the differentiation of T cells into proinflammatory type 1 helper T (Th1) cells and monocytes into macrophages. This process subsequently enables downstream inflammatory responses [13, 37, 42]. Several pathways have been proposed to explain the impact of CCL14 on renal function. First, CCL14 may promote inflammation and fibrosis [42–44], which can impair renal function. Second, CCL14 may cause immune modulation, which leading to an inadequate response to the injury [40]. Lastly, CCL14 may trigger the apoptosis of renal tubular cells, further damaging the kidney given that it showed the property of cell cycle modulation and promoting apoptosis in vitro [45]. As CCL14 plays a role in mediating inflammatory and profibrotic pathways, its elevated levels may indicate persistent renal dysfunction following AKI episodes, as shown in previous clinical studies [17, 24, 25].

In the current meta-analysis, the diagnostic thresholds of urinary CCL14 varied among the enrolled studies yet all of them demonstrated high diagnostic sensitivity and good negative predictive value. On the other hand, the enrolled studies showed considerable variation in diagnostic specificity and positive predictive value, and the combined results were only moderately satisfactory. These results suggest that urinary CCL14 may exhibit greater effectiveness in clinical settings characterized by a higher risk of persistent AKI, in contrast to those with a lower risk. In critical care or surgical settings, the potential advantages of reducing kidney injury-related complications may outweigh any potential harms associated with excessive monitoring [29, 46–48]. Consequently, urinary CCL14 shows great promise as a useful biomarker for such patients.

The results of this study may provide clinical practitioners and researchers with important information regarding the clinical application of urinary CCL14 and future research directions in this area. By improving our understanding of the predictive performance of urinary CCL14 for persistent AKI, we might have the potential to improve patient outcomes by enabling prompt intervention and more effective management of AKI.

### Limitation

While our study yielded promising outcomes, it is important to acknowledge several important limitations. First, the meta-analysis was hindered by the small sample sizes of most studies, leading to high heterogeneity. Furthermore, while the majority of studies enrolled patients with stage 2 or 3 AKI, two studies included a substantial number of patients with stage 1 AKI or non-AKI [19, 24]. This heterogeneity of the patient cohorts in the included studies further contributed to the overall heterogeneity of the results. While there was no significant publication bias as shown by the funnel plot and Cochrane Collaboration tool analysis, the limited number of studies made it impossible to conduct subgroup analysis. Second, the studies included in the meta-analyses were all observational in nature and did not account for potential confounding factors. Furthermore, we were unable to include the secondary analysis study of SAPPHIRE conducted by Bagshaw et al. due to data limitations. Third, all the included studies were conducted in critically ill or surgical patients, which limits the generalizability of our findings to other clinical settings. Fourth, we were unable to compare the predictive performance of urinary CCL14 and the traditional methods currently used in clinical practice for predicting persistent AKI, as the enrolled studies did not provide related predictive information about serum creatinine (SCr). However, SCr levels are known to have limited predictive performance for AKI due to their delayed rise, inability to accurately estimate the timing of injury, and susceptibility to fluid status influences. Therefore, there are limitations to using SCr as a comparison, including the potential for both overestimating and underestimating the predictive performance of biomarkers. Moreover, in the studies by Massoth et al. and Koyner et al. [24, 25], the inclusion of urinary CCL14 in models constructed using clinical parameters to predict severe persistent AKI can improve the predictive ability of the models. Furthermore, in Koyner’s study, they also conducted multivariable adjusted analysis and found that even after adjusting for other clinical factors, urinary CCL14 still possesses remarked predictive ability for persistent severe AKI. However, the limited number of current studies focusing on these specific evaluations regarding the predictive significance of urinary CCL14 presents a challenge in performing a meta-analysis on this subject. The future emergence of more relevant research is necessary to further validate these particular aspects. Fifth, the kits for CCL14 analysis varied among studies, making it difficult to determine the optimal cutoff value of urinary CCL14 to predict persistent AKI. Sixth, all six studies included in our analysis were observational, and as such, the findings should be interpreted with caution. It is crucial to note that the certainty of evidence for our findings was initially rated low due to the observational nature of the studies included. Although the majority of the studies were deemed to be of high quality, with only two rated as moderate, the possibility of selection bias, information bias, and confounding factors may still have influenced our results. Seventh, it should be noted that this meta-analysis exhibits heterogeneity in both pooled sensitivity and pooled specificity, which could be due to variations in study design, baseline characteristics, timing of urinary CCL14 measurement, and the kits used for CCL14 level analysis. These factors may have impacted the overall accuracy of the results, thus reducing the confidence level of the present findings. In addition, the definition of persistent AKI also varied among the studies, potentially impacting the pooled effect estimates. Specifically, one study had a particular focus on critically ill patients who required RRT. Nonetheless, our findings demonstrated consistency and robustness in sensitivity analyses, as the results remained unaffected by the exclusion of any individual study. Despite the aforementioned limitations, the conclusions drawn from our study were based on a range of studies with varying designs and clinical contexts. To further advance precision medicine, future research efforts should explore whether the predictive accuracy of urinary CCL14 is impacted by the specific etiology of AKI and the severity of the condition. These issues could be incorporated into future randomized controlled trials, which could help to identify the optimal cutoff values for different clinical settings and improve the timely diagnosis and management of persistent AKI. Additionally, further investigations into the underlying mechanisms of AKI may aid in enhancing predictive performance and timely treatment, potentially reducing the high mortality rate among AKI patients.

## Conclusion

In conclusion, the findings of this systematic review suggest that urinary CCL14 may serve as a potential biomarker for predicting persistent AKI in critically ill patients or those undergoing cardiac surgery. However, further research is required to establish a distinct diagnostic threshold for urinary CCL14 to enhance its clinical utility in predicting persistent AKI. While the meta-analysis demonstrated good overall accuracy, the limitations and variations in the included studies highlight the need for additional clinical trials and real-world data to validate the use of urinary CCL14 as a biomarker for predicting persistent AKI.

### Supplementary Information

**Additional file 1**. Supplementary appendix.

## Data Availability

The datasets used and/or analyzed during the current study are available from the corresponding author on reasonable request.

## Abbreviations

AKI: Acute kidney injury
CCL14: C–C motif chemokine ligand 14
CKD: Chronic kidney disease
COE: Certainty of evidence
ESRD: End-stage renal disease
FP: False positives
FN: False negatives
HCC-1: Human CC chemokines-1
HSROC: Hierarchical summary receiver operating characteristic curves
KDIGO: Kidney disease: improving global outcomes
PRISMA: Preferred reporting items of systematic reviews and meta-analyses
QUADAS-2: Quality assessment of diagnostic accuracy studies-2
RRT: Renal replacement therapy
TP: True positives
TN: True negatives
SCr: Serum creatinine
