# Supplementary material for: Performance of urinary C–C motif chemokine ligand 14 for the prediction of persistent acute kidney injury: a systematic review and meta-analysis
Source: Crit Care. 2023 Aug 18;27:318. doi: 10.1186/s13054-023-04610-7 (PMC10439656; doi:10.1186/s13054-023-04610-7)

**Supplementary appendix**

**This supplementary appendix provides:**

1. **Search equation via PubMed, EMBASE, MEDLINE, and** [**Cochrane**](http://www.cochranelibrary.com/) **library**
2. **Quality assessment of the included studies**
3. **PRISMA checklist**
4. **Other supplemental Figures**
5. **Summary of contextual factor data**
6. **PROSPERO protocol registration**
7. **The GRADE results**
8. **Search equation via PubMed, EMBASE, MEDLINE, and Cochrane library**

Search strategies for the different databases ran on **April 12, 2023**

**PubMed (7)**

"CCL14"[All Fields] AND (("predict"[All Fields] OR "predictabilities"[All Fields] OR "predictability"[All Fields] OR "predictable"[All Fields] OR "predictably"[All Fields] OR "predicted"[All Fields] OR "predicting"[All Fields] OR "prediction"[All Fields] OR "predictions"[All Fields] OR "predictive"[All Fields] OR "predictively"[All Fields] OR "predictiveness"[All Fields] OR "predictives"[All Fields] OR "predictivities"[All Fields] OR "predictivity"[All Fields] OR "predicts"[All Fields]) AND (("acute kidney injury"[MeSH Terms] OR ("acute"[All Fields] AND "kidney"[All Fields] AND "injury"[All Fields]) OR "acute kidney injury"[All Fields]) AND ("biomarker s"[All Fields] OR "biomarkers"[MeSH Terms] OR "biomarkers"[All Fields] OR "biomarker"[All Fields])))

**Embase (19)**

('acute kidney injury'/exp OR 'acute kidney injury' OR (acute AND ('kidney'/exp OR kidney) AND ('injury'/exp OR injury))) AND 'c c' AND motif AND chemokine AND ligand AND 14

**Medline (5)**

(CCL14 and (prediction and (acute kidney injury and biomarkers))).mp. [mp=title, book title, abstract, original title, name of substance word, subject heading word, floating sub-heading word, keyword heading word, organism supplementary concept word, protocol supplementary concept word, rare disease supplementary concept word, unique identifier, synonyms, population supplementary concept word, anatomy supplementary concept word]

**Cochrane library (2)**

#1 C-C motif chemokine ligand 14

#2 acute kidney injury

#3 #1 AND #2

1. **Quality assessment of the included studies**

**Supplemental Figure 1**: **Risk of bias and applicability concerns using the QUADAS-2 tool for each included study based on the complete literature-based analysis**

**Supplemental Figure 2. Overview about the risk of bias and applicability concerns graph: using the QUADAS-2 tool based on the complete literature-based analysis**

1. **
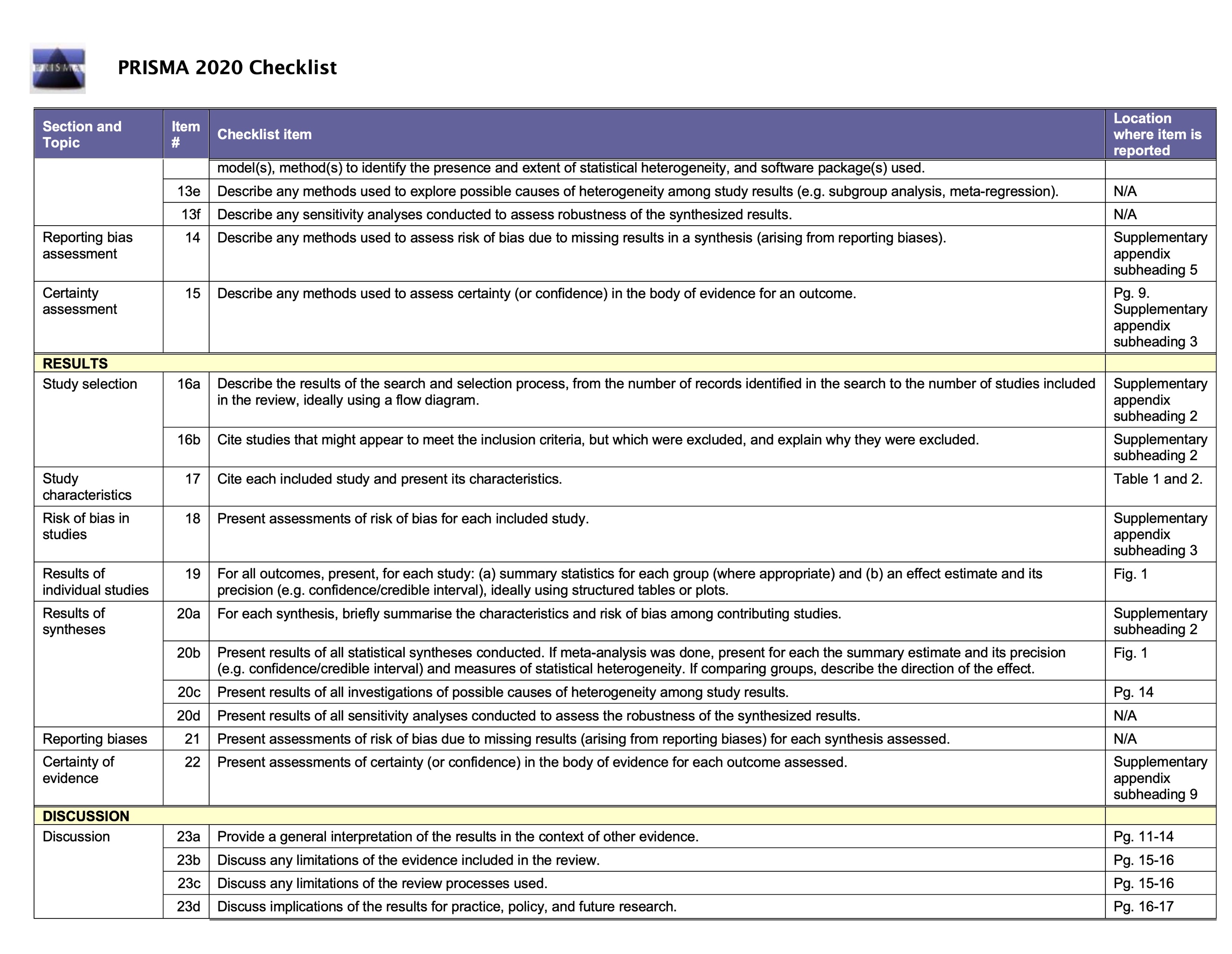

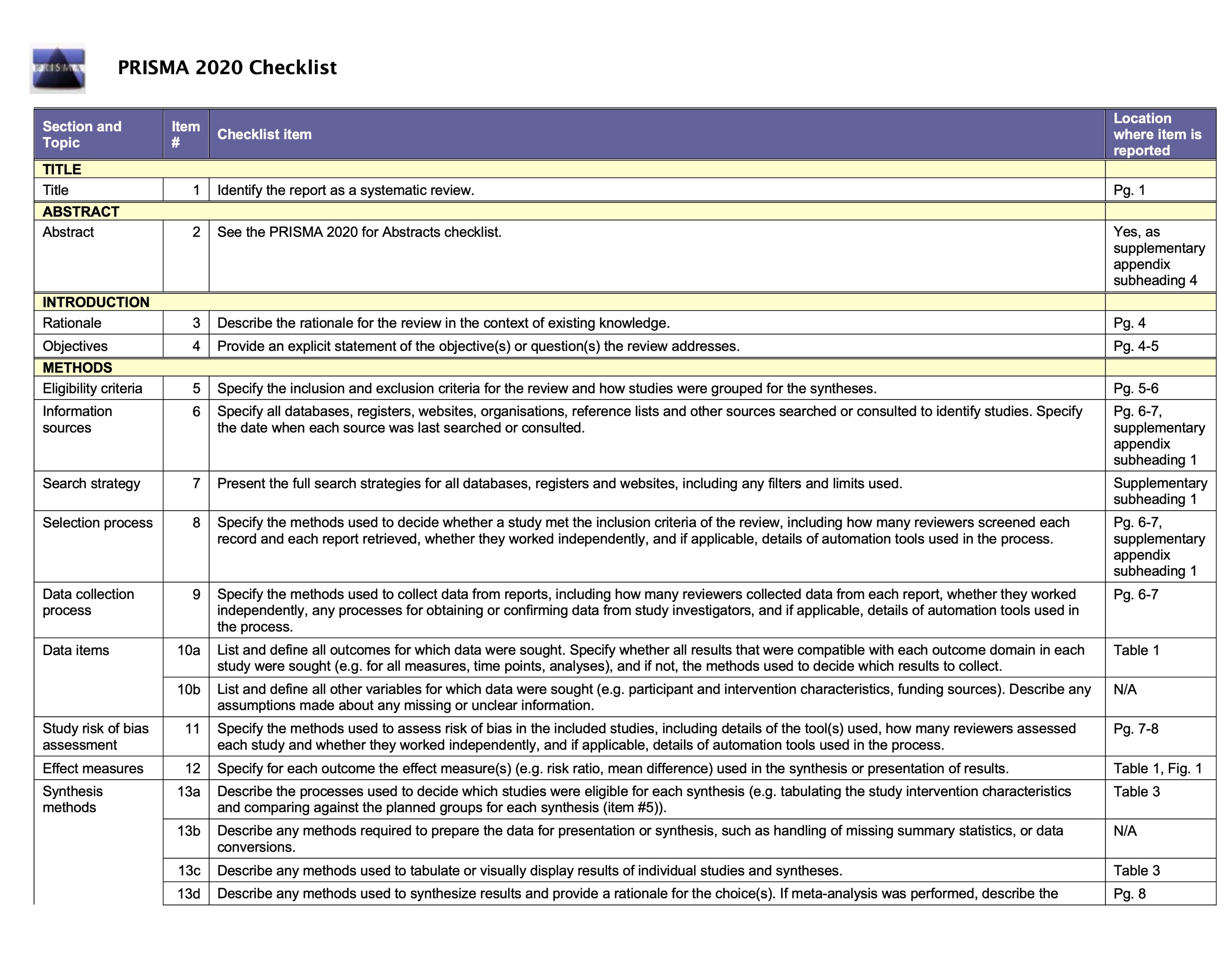
PRISMA checklist**

**
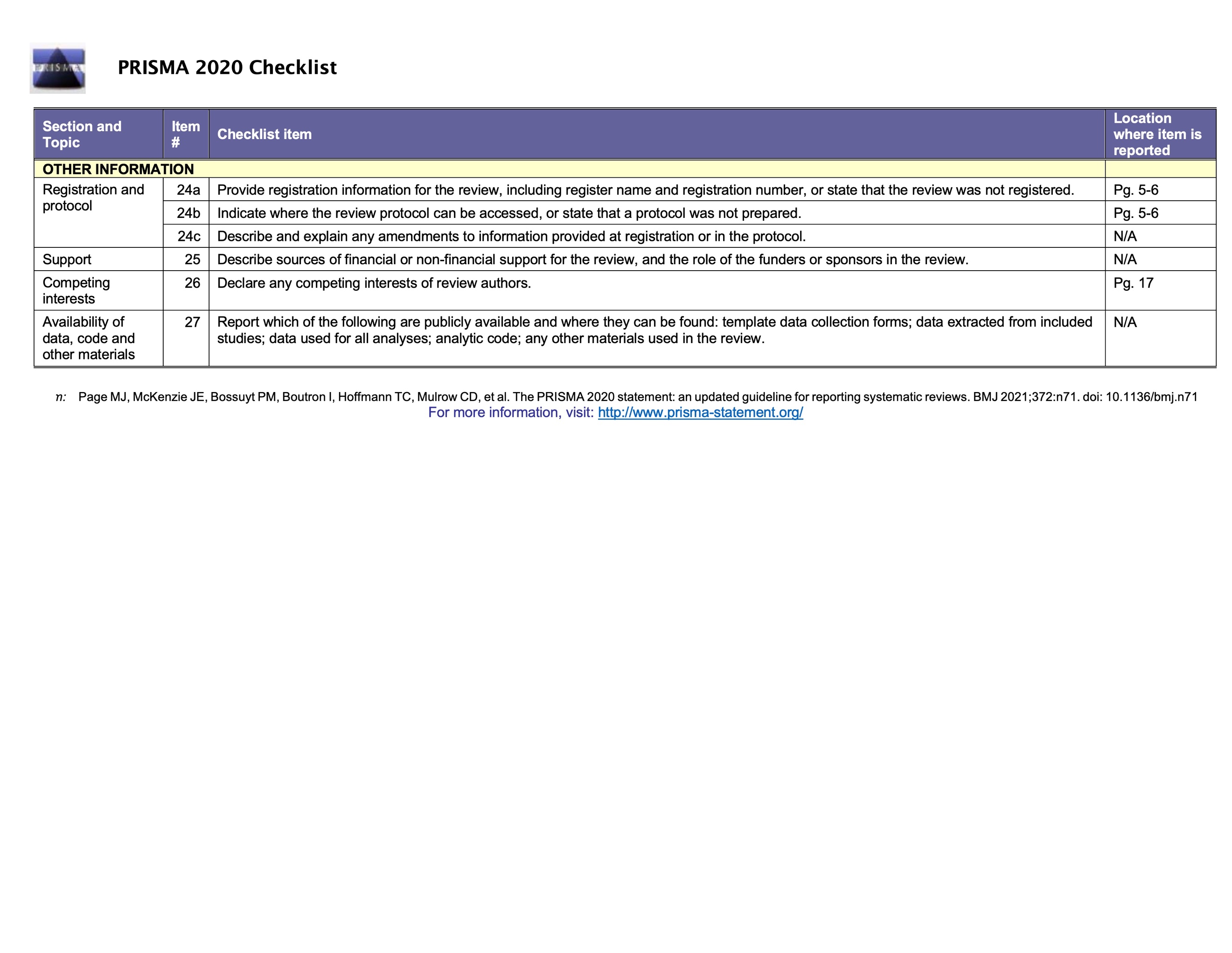

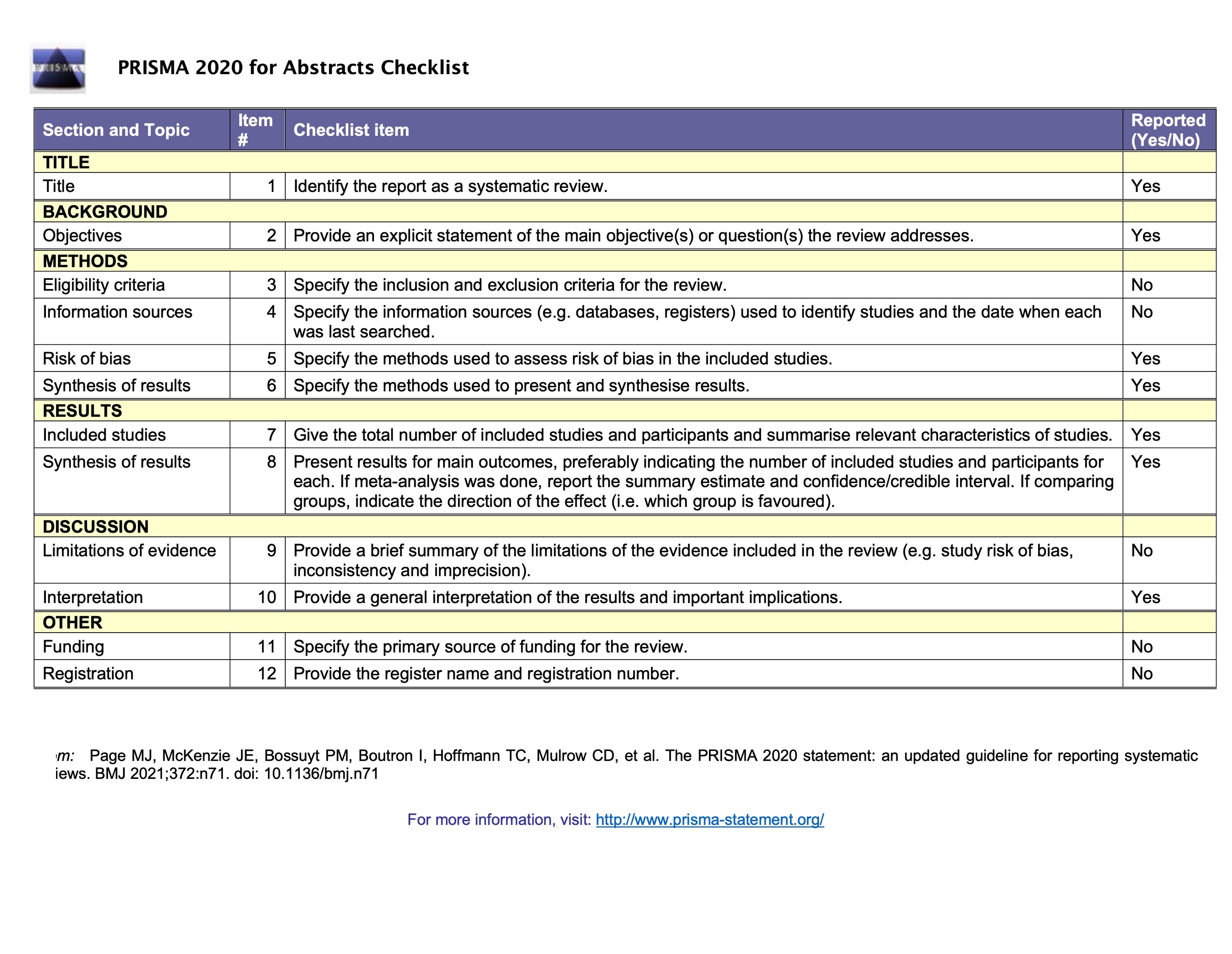
**

**
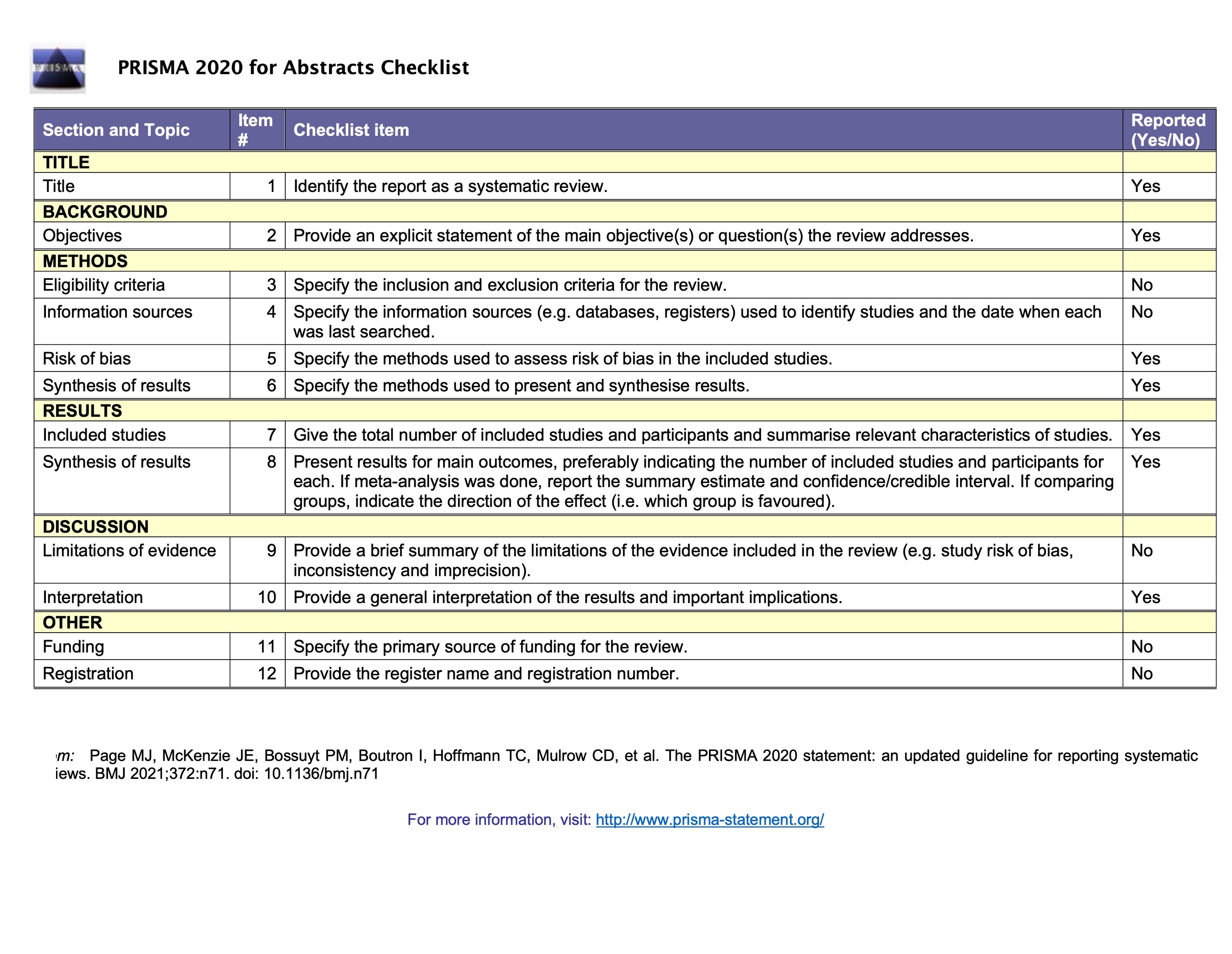
 Prisma check list for abstract**

1. **Other Supplemental Figures**

**Supplemental Figure 3. The Fagan nomogram for the performance of CCL-14 in predicting persistent AKI.**

1. **With a pretest probability of 39.6% for persistent AKI**

1. **With a pretest probability of 25% for persistent AKI**

1. **With a pretest probability of 75% for persistent AKI**

**Supplemental Figure 4. Deek’s funnel plot for the performance of CCL-14 in predicting persistent AKI.**

**Supplemental Figure 5. Accuracy of CCL14 for prediction of persistent AKI (excluding Pan’s study).**

**Abbreviations:** AKI, acute kidney injury; CCL14, C–C Motif Chemokine Ligand 14; HSROC, hierarchical Summary Receiver Operating Characteristic.

**Supplemental Figure 6. The forest plot for evaluating the pooled sensitivity of CCL-14 in predicting persistent AKI (excluding Pan’s study).**

**Abbreviations:** AKI, acute kidney injury; CCL14, C–C Motif Chemokine Ligand 14; CI, confidence interval.

**Supplemental Figure 7. The positive and negative likelihood ratios of CCL-14 predictive accuracy for persistent AKI (excluding Pan’s study).**

**Abbreviations:** AKI, acute kidney injury; CCL14, C–C Motif Chemokine Ligand 14; LR, likelihood ratio; NPV, negative predictive value; [PPV, positive predictive value.](https://www.ncbi.nlm.nih.gov/pmc/articles/PMC7845419/)

**Supplemental Figure 8. Graphic abstract**


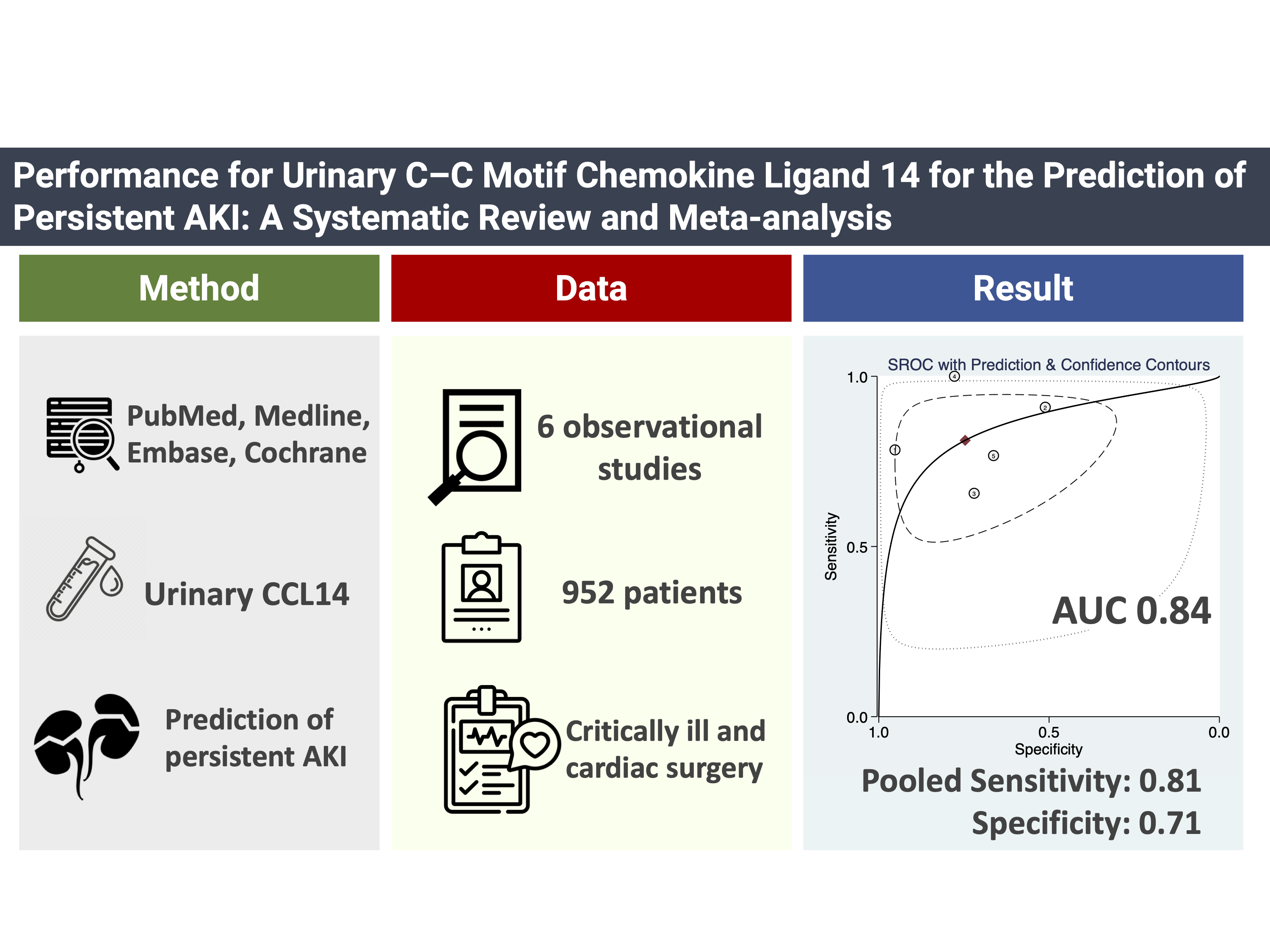


**Abbreviations:** AKI, acute kidney injury; CCL14, C–C Motif Chemokine Ligand 14.

1. **Summary of contextual factor data**

**For analysis of the diagnostic performance of CCL14 in predicting persistent AKI, 6 observational studies, comprising 952 participants with complete data and target endpoints, were included in the final meta-analysis.**

**Massoth et al (2021)** included 100 patients undergoing cardiac surgery who developed stage 2-3 AKI. According to their findings, setting the CCL14 cutoff value at 2.21 ng/mL resulted in a sensitivity of 78% and a specificity of 95%. Urinary CCL14 was able to predict the primary end point with an AUC of 0.93 (95% CI, 0.881-0.979)

**Koyner et al (2022)**, **a secondary analysis from RUBY study,** included 335 critically ill patients who diagnosed with stage 2-3 AKI. They defined a CCL14 cutoff value of 1.3 ng/ml to achieve a sensitivity 91% (84-96%), specificity 51% (44-57%), positive predictive value 47% (40-54%) and negative predictive value 92% (86-96%) for identifying persistent severe AKI. The AUC of urine CCL14 for predicting the persistent severe AKI is 0.82 (95% CI, 0.77-0.87).

**Pan et al (2022)** included 140 critically ill patients with AKI requiring dialysis. Based on the data provided by the author, a cutoff value of 1.4 ng/ml for urine CCL14 was identified to achieve a sensitivity of 0.81 and specificity of 0.49 for predicting persistent AKI, which is characterized by dependence on renal replacement therapy, with an AUC of 0.67.

**Jiang et al (2023)** included 48 critically ill patients who diagnosed with AKI. Among them, 23 developed persistent AKI (AKI >=48 hrs). The subgroup analysis (total 18 patients) with more severe AKI (KDIGO stage 2 and 3) showed a cutoff value of 0.603 ng/ml resulting a sensitivity 100% and specificity 78% for predicting persistent and severe AKI with AUC 0.85 (95% CI, 0.64-1.00)

**Qian et al (2023)** included 164 critically ill patients. In their cohort, CCL14 had predictive value for renal non-recovery with an AUC of 0.71 (95% CI, 0.63-0.77). They determined that an optimal cutoff value of 625.69 pg/ml resulted in a sensitivity of 65.6% and a specificity of 72.0%.

**Meersch et al (2023)** included 208 critically ill patients. In their subgroup analysis (case number=195), CCL14 had predictive value for stage 3 AKI with an AUC of 0.75. They determined that an optimal cutoff value of 2.44 ng/ml resulted in a sensitivity of 71.8% and a specificity of 66.3%.

1. **PROSPERO protocol registration**

**
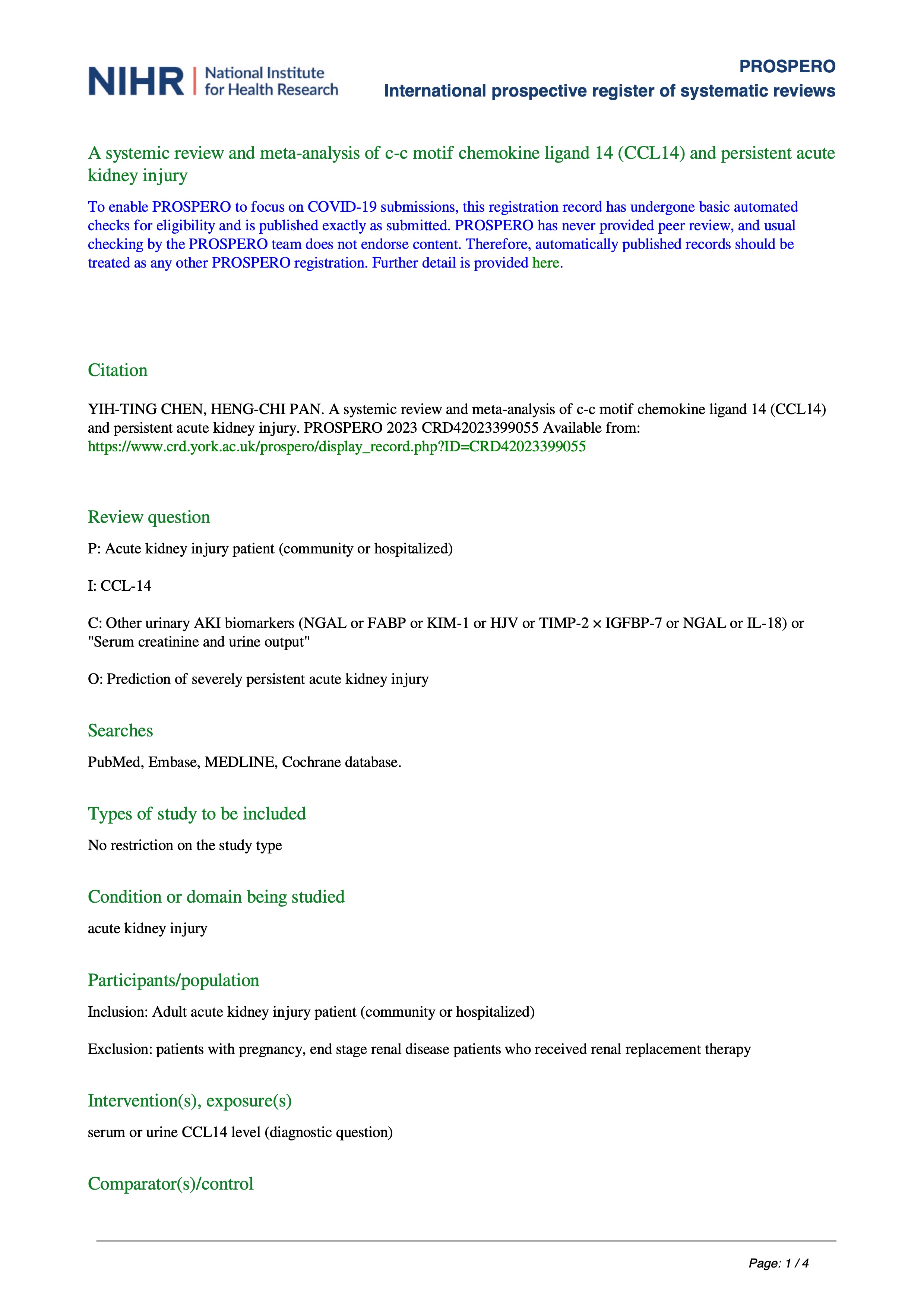
**PROSPERO 2023 CRD42023399055

**
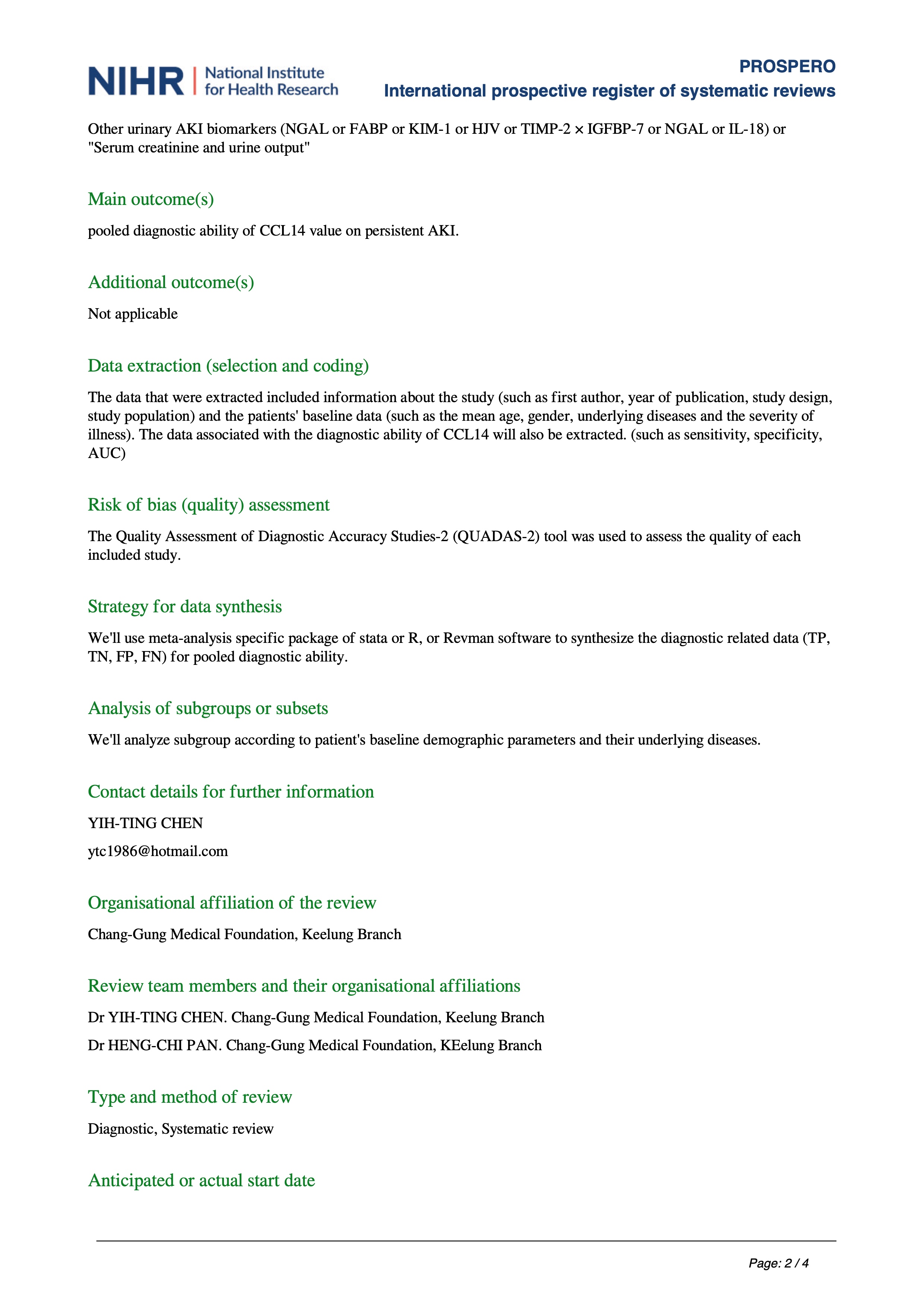
**

**
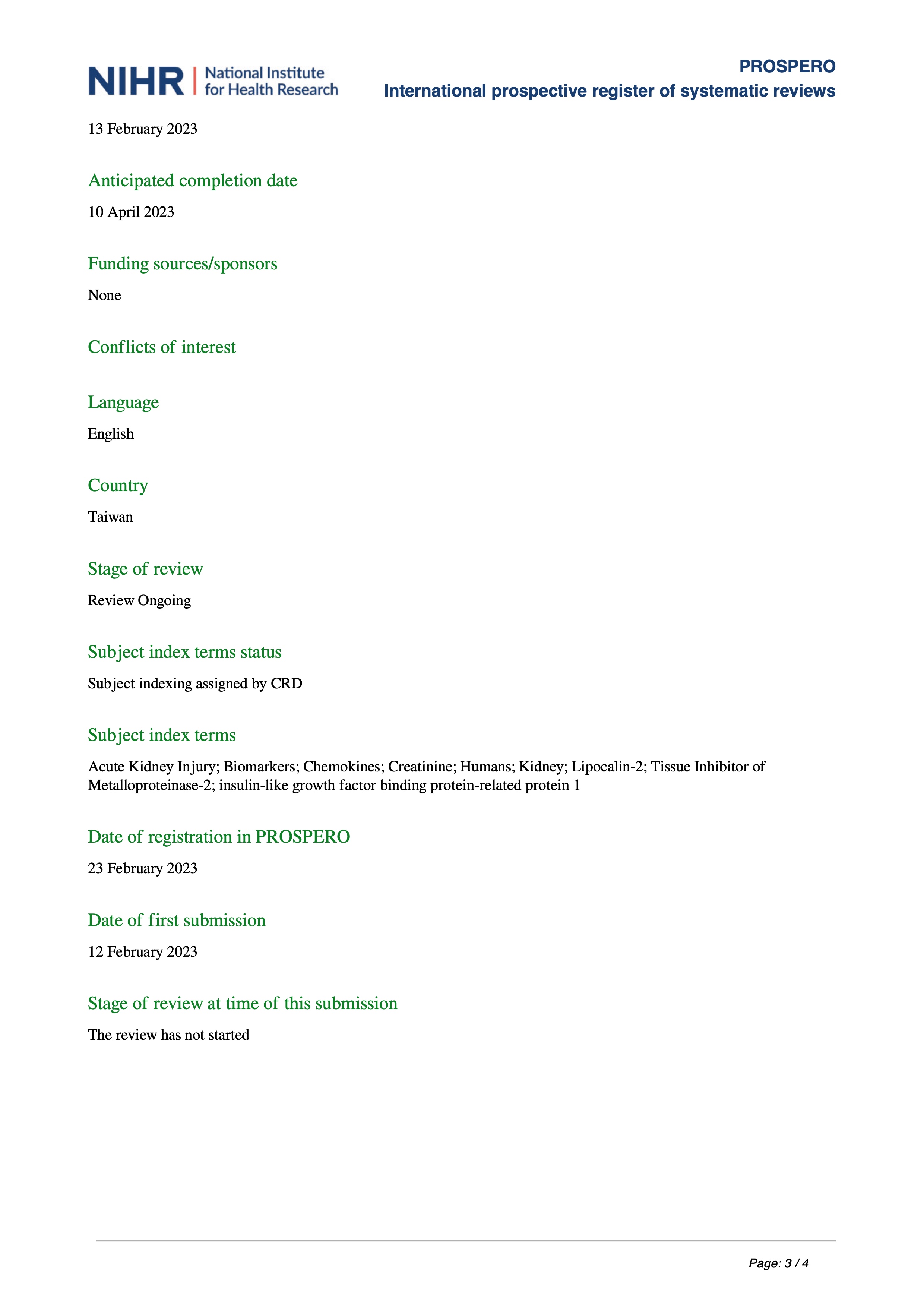
**

**
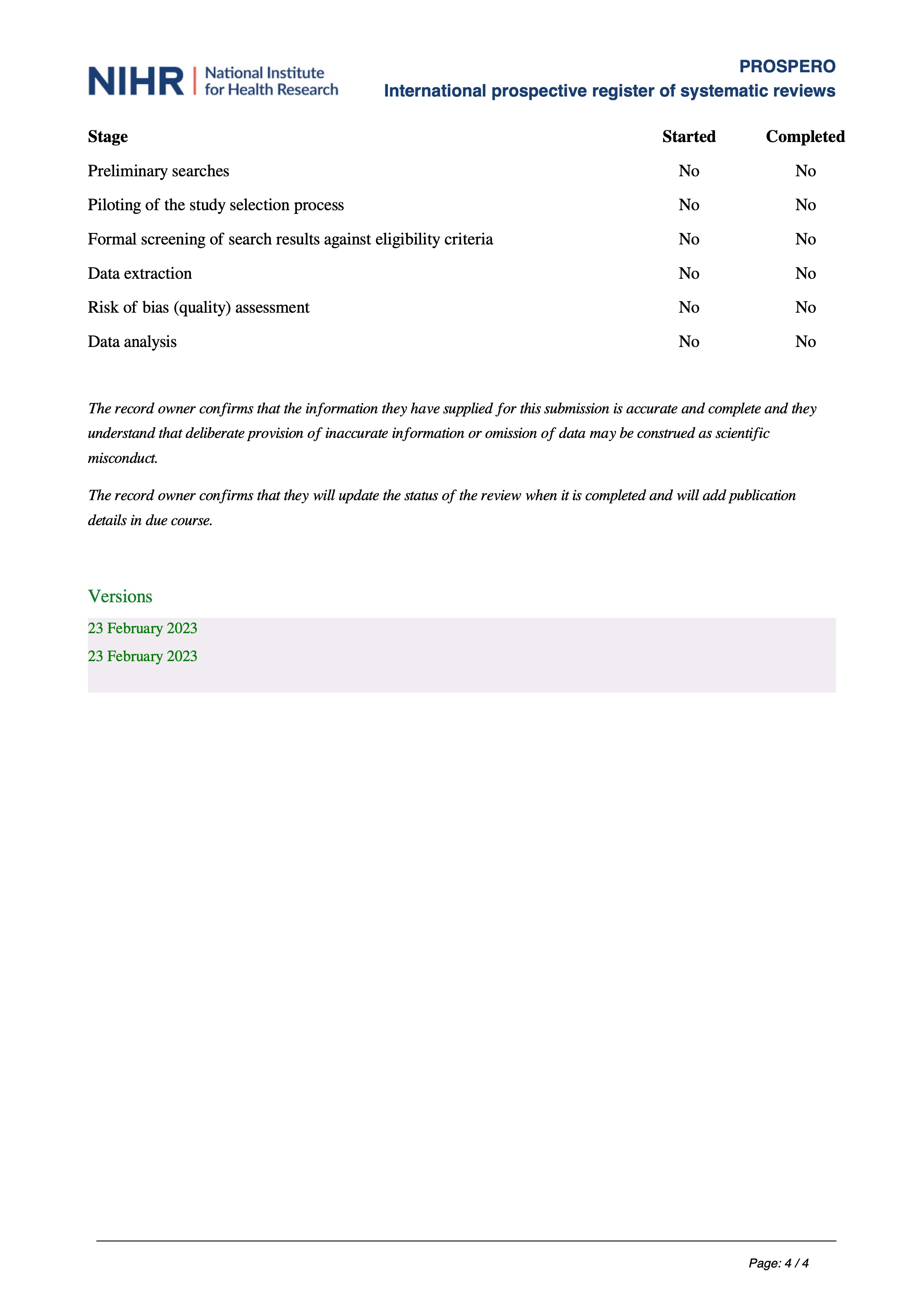
**

1. **The GRADE results**


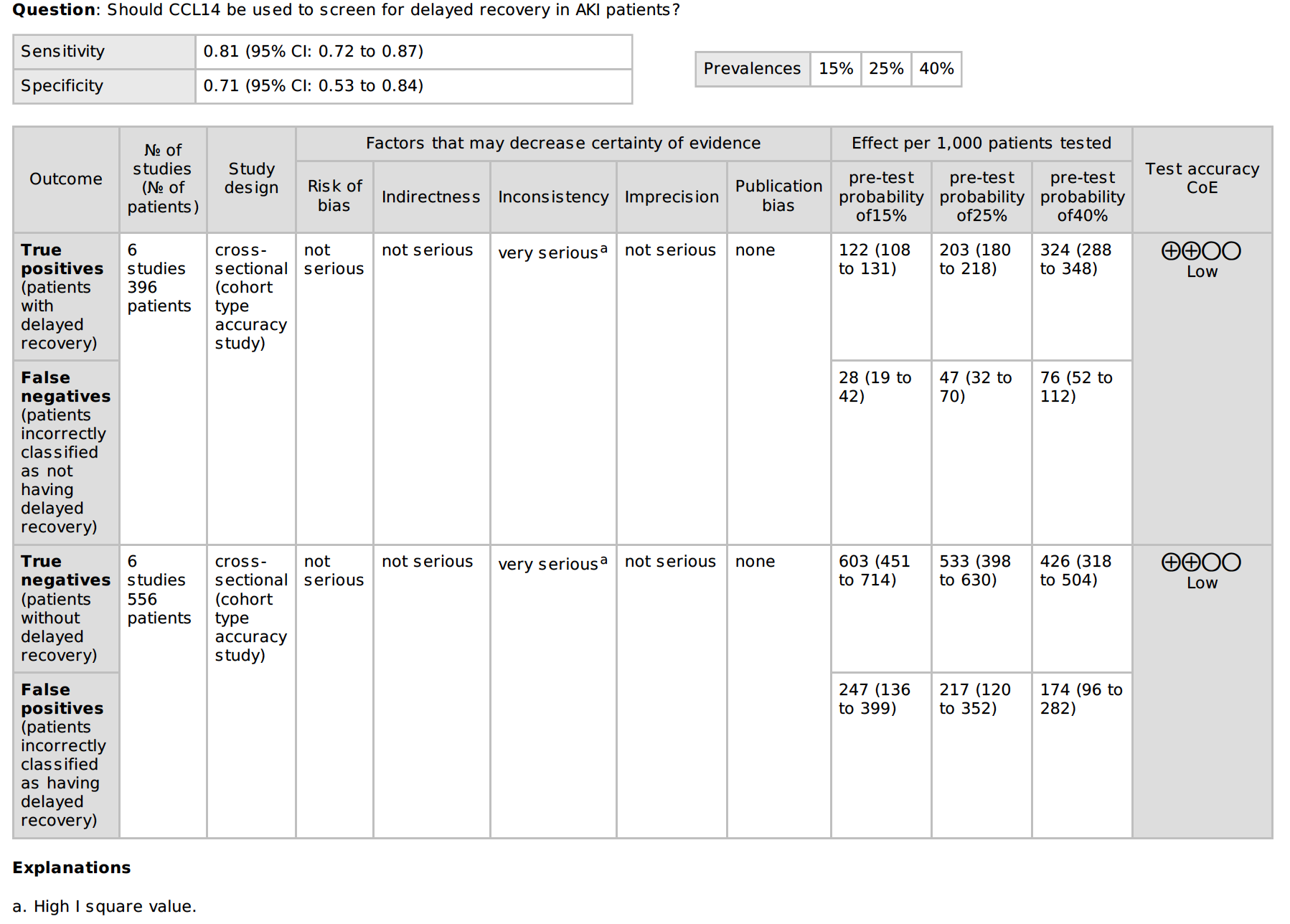

Supplement: Supplementary file 1 — Additional file 1. Supplementary appendix. [file 13054_2023_4610_MOESM1_ESM.docx]
